# Toxicity, Antibacterial, and Phytochemical Analyses of *Antrocaryon klaineanum* Pierre Extracts

**DOI:** 10.1155/2023/9304681

**Published:** 2023-04-22

**Authors:** Cédric Sima Obiang, Thiery Ndong Mba, Joseph Privat Ondo, Rick Léonid Ngoua Meye Misso, Juliette Ornely Orango Bourdette, Elvis Otogo N'Nang, Joefred Mbogho Abogho, Elvis Jolinom Mbot, Louis Clément Obame Engonga, Edouard Nsi Emvo

**Affiliations:** ^1^Laboratoire de Recherche en Biochimie (LAREBIO), Faculté des Sciences, Université des Sciences et Techniques de Masuku, Franceville, Gabon; ^2^Laboratoire de Substances Naturelles et de Synthèses Organométalliques (LASNSOM), Université des Sciences et Techniques de Masuku, Franceville, Gabon; ^3^Laboratoire de Biologie Moléculaire et Cellulaire (LABMC), Faculté des Sciences Université des Sciences et Techniques de Masuku, Franceville, Gabon; ^4^Laboratoire de Chimie des Substances Naturelles (LACSN), Institut Supérieure d'Agronomie et de Biotechnologie, Université des Sciences et Techniques de Masuku, Franceville, Gabon

## Abstract

Medicinal plants are traditionally used in Gabon to treat several types of illnesses. The study's purpose was to determine the toxic, antibacterial, and anti-inflammatory effects of *Antrocaryon klaineanum* Pierre extracts and to characterize their phytochemical compounds. Toxicity was evaluated on frog tadpoles (*Phrynobatrachus africanus* Hallowell). The microorganism susceptibility test was performed by the diffusion method, while minimum inhibitory concentration (MIC) and minimum bactericidal concentration (MBC) were evaluated using the microdilution technique. Anti-inflammatory activity was tested through protein denaturation and membrane stabilization methods. Chromatography and molecular network techniques were used to characterize chemical compounds. The lethality test showed that the lethal concentration (LC_50_) increased from 110.03 ± 1.25 to 15.86 ± 2.21 *μ*g/mL after 24 and 96 hours of exposure. In tadpoles exposed to 7.81 *μ*g/mL extract, the first mortalities (12.5%) were observed on the fifth day of exposure. A relative decrease in mature erythrocytes exposed to plant extracts was observed. The antibacterial activity shows that the Ak F_2_, Ak F_3_, and Ak F_4_ fractions (from the water-ethanol crude extract) gave the greatest antibacterial activities compared to the other extracts. The water, water-acetone, and water-ethanol extracts showed good inhibition of denaturation. The haemolysis test shows that the extracts exhibited good anti-inflammatory activities. Phytochemical characterisation revealed four major compounds, including monogallate epicatechin and hydroxy-ergostadian. The molecular network revealed five main clusters. Our study shows that *A. klaineanum* Pierre could be a promising natural product for the isolation of molecules with potential biological activities.

## 1. Introduction

Traditional medicine is often the only affordable and accessible source of care in poor countries, especially for the poorest patients. According to the World Health Organisation (WHO), more than 80% of the African population regularly uses traditional and herbal medicine for the treatment of various conditions [1]. Plant research can lead to acceptable therapeutic responses and low-cost pricing in a socioeconomic environment, combining proven scientific efficacy with ideal cultural acceptability. The scientific validation of traditional medicine should contribute to the development of phytomedicines.

Given the scientific interest in plant-based medicines, recent years have seen the therapeutic valorisation of biologically active molecules contained in plant species. This has resulted in the creation of new molecules of natural origin. Despite the large amount of research that has already been carried out on plants, the results are still insufficient, given that only a small number of plants have been systematically studied for bioactive compounds [2].

Many researchers are interested in biologically active compounds isolated from plant species for the elimination of pathogenic microorganisms because of the resistance that microorganisms have developed against synthetic antibiotics. Gabon, by virtue of its geographical location, is home to a rich and diverse vegetation.

Many studies, albeit scattered, have been carried out on *A. klaineanum*, which shows the importance attached to traditional medicine. *Antrocaryon klaineanum* Pierre (Family: Anacardiaceae) is known by several names in Gabon, depending on the ethnic group (Angokong (Fang), Osôngôngô (Mpongwè); mungôngu-bôgu (Bapunu), and mungôngu-bôgu (Banzabi)); in Cameroon its common name is Onzabili [3, 4]. It is a very large tree that is common throughout Gabon. It has compound leaves. The flowers are yellowish-white. The fruits are flattened and pentagonal, with a very hard, evenly shaped stone. The powder of the bark is used against liver diseases [3], it also treats liver diseases. The roots treat abdominal and liver diseases [5]. In Cameroon, its bark is used in traditional medicine to treat wounds, chlamydia, and female sterility [6]. Phytochemical studies on the isolation of Antrocarins A-F of the ergostane steroid type and antiplasmodial activities were carried out by Douanla [7]. Other works have shown that *A. klaineanum* has antioxidant and anti-inflammatory properties [8, 9].

However, research on toxicity and other biological properties of *A. klaineanum* stem barks is limited. The present context aims not only to study the toxicity, antibacterial, and anti-inflammatory properties but also to characterise the phytochemical constituents of *A. klaineanum*.

## 2. Material and Methods

### 2.1. Chemicals, Reagents, and Media

Organic solvents (HPLC grade) were obtained from Himedia, India. NaCl (Suvchem), sodium chloride (NaCl); Potassium chloride (KCl); Calcium chloride (CaCl_2_); agar (Biokar); glucose (Accumix); and other chemicals were obtained from Sigma, USA. All chemicals used were of analytical grade.

### 2.2. Preparation of Samples

The stem bark of *A. klaineanum* (voucher number: AK01-2017) was collected in Oyem (northern Gabon), in June 2017 and identified at the National Herbarium by Professor Henry Bourobou (Botanist). The harvested bark was dried, crushed, and used for extractions.

The water-ethanol (30/70, v/v), water-acetone (30/70, v/v), and water (100%) extracts were prepared from the dry powder of *A. klaineanum*. Each sample (25 g) was mixed with 250 mL of extraction solvents. The aqueous extracts were boiled for 60 minutes; water-ethanol and water-acetone extracts were macerated for 24 h. All extracts were filtered, concentrated, and lyophilized. The extracts obtained were stored at 4°C until they were used for the various tests.

### 2.3. Toxicity of Aqueous Extracts on Frog Tadpoles (*Phrynobatrachus africanus* Hallowell)

#### 2.3.1. Tadpole Sampling

The determination of *Phrynobatrachus africanus* Hallowell was carried out by the identification guide of amphibians of central and southern Africa [10]. The tadpoles of this species were captured in June 2021 in a body of water near a river. Upon their arrival at the laboratory, the tadpoles were placed in glass basins containing reconstituted water. The tadpoles selected for the study had a mass of 2.00 ± 1.00 g and were at a stage of development corresponding to the appearance and progression of the buds of the hind limbs.

#### 2.3.2. Lethal and Sublethal Testing

Lethal and sublethal tests were performed using the method described by Obiang et al. [11].

#### 2.3.3. Hematological Parameters

After 1, 4, 8, and 16 days of exposure, the tadpoles were sacrificed according to the concentrations (1.95; 3.9; 7.81 *μ*g/mL) of the extract in order to collect the biological material necessary for the hematological analyses. The tadpoles were first anesthetized and 5 *μ*L of blood was collected by cardiac puncture. A smear was immediately prepared, followed by Leishman staining, then the cells were observed under an optical microscope (Motic Digital Microscope) coupled to a computer using Motic image plus 2.0 software, and finally photos were taken.

*(1) Relative Proportions of Erythrocytes*. To determine the relative proportions of erythrocytes, 500 cells were identified and categorized into three subgroups: mature erythrocytes, erythroblasts, and degenerating erythrocytes. The erythroblasts were recognizable, according to the description made by Szubartowska [12], by their rounded shape, basophilic cytoplasm, and prominent nucleus, while degenerating erythrocytes were identified by their wrinkled and stunted appearance. The percentage of mature erythrocytes, erythroblasts, and degenerating erythrocytes per 500 counted cells (total erythrocytes) per individual was determined.

### 2.4. Bacterial Germs Tested

The bacterial carrier used in our study consisted of six reference bacterial strains and four clinical strains.

### 2.5. Antibacterial Activities

The diffusion method was used to study the susceptibility of the microorganisms. Minimum inhibitory concentrations (MICs) of crude extracts and fractions were determined by the microdilution method in 96-well microplates [13]. Bactericidal antibacterials were considered to be those with a MBC/MIC ratio of 1 or 2 and bacteriostatic if the MBC/MIC ratio was 4 or 16 [14].

### 2.6. Anti-Inflammatory Activities

#### 2.6.1. Antiprotein Denaturation Test

Denaturation inhibition of crude extracts and fractions of *Antrocaryon klaineanum* was performed according to the protein denaturation inhibition method described in the reference by Ngoua-Meye-Misso et al. [15]. The references (diclofenac sodium and paracetamol) used were treated under the same conditions as the crude extracts.

#### 2.6.2. Membrane Stabilization Test

The membrane stabilisation test was evaluated by the human red blood cell (HRM) haemolysis method. This haemolysis was induced by heat on the one hand and distilled water on the other [15].

### 2.7. Fractionation, Identification, and Molecular Network Analysis

Flash chromatography is a method of separation based on polarity; it has the same process as column chromatography. Separation by flash chromatography was carried out on Armen Instrument, spot liquid chromatography flash, according to the gradient of increasing polarity. 32 g of the stem bark water-alcohol extract was submitted to flash chromatography on a 70–230 mesh silica gel column (400 g) with stepwise gradient elution by CH_2_Cl_2_/MeOH mixtures (100 : 0; 98 : 2; 95 : 5; 90 : 10; 80 : 20; 0 : 100). The fractions were collected and combined according to their thin layer chromatography (TLC) profiles on precoated silica gel 60 F_254_ plates developed with n-hexane/EtOAc and CH_2_Cl_2_/MeOH mixtures to give groups of fractions. The fraction with the major biological activities was selected and analysed by high performance liquid chromatography coupled with the mass spectrometry (HPLC/Q tof). The compounds obtained were identified by comparisons of their mass spectra to those of compounds registered in Reaxis and DNP 2019 libraries.

Molecular networking was carried out as described by Essono Mintsa et al. [16].

### 2.8. Statistical Analysis

The data were expressed as mean ± standard deviation (SD) of triplicate independent experiments and analyzed using one-way analysis of variance (ANOVA) and Student's *t*-test using Staplus Build software. 8.03/Corev7.811 (x86_64). The values of *p* ≤ 0.05 were considered statistically significant.

## 3. Results

### 3.1. Toxicity of Aqueous Extracts of *Antrocaryon klaineanum* Pierre

#### 3.1.1. Lethal Test

The results of the analyses presented in Table 1 show that the LC_50_ for frog tadpoles exposed to aqueous extracts of *A. klaineanum* decreases proportionally to the days.

This LC_50_ is 110.03 ± 1.25 (APB), 63.55 ± 4.02 (APB), 29.36 ± 2.11 (APB), and 15.86 ± 2.21 (APB) *μ*g/mL after 24, 48, 72, and 96 hours of tadpole exposure, respectively. The decrease in the value of LC_50_ results in the evolution of the increasing number of mortalities. After 96 hours of exposure, no tadpole survived the other concentration ranges of 15.62, 31.2, 62.5, 125, 250, 500, and 1000 *μ*g/mL when all were alive in tanks containing only reconstituted water and the lowest concentrations of aqueous extracts of *A. klaineanum*.

#### 3.1.2. Mortalities during Sublethal Tests

**Figure 1:**
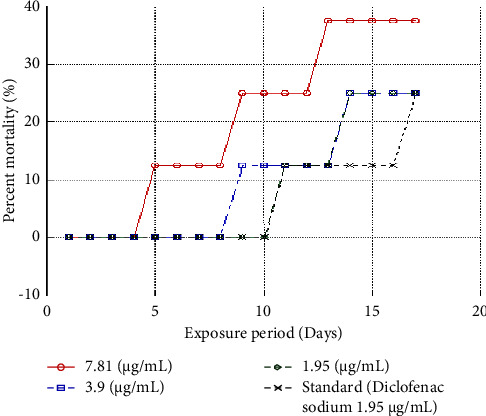
Mortalities recorded during the sublethal test in tadpoles exposed to aqueous extract of *Antrocaryon klaineanum* for 17 days.

Figure 1 presents the mortalities recorded during the sublethal test in tadpoles exposed to extracts of *A. klaineanum* for 17 days.

The result shows the first mortalities (12.5%) of the tadpoles on the fifth day at 7.81 *μ*g/mL of the plant extracts. On the ninth and thirteenth days, the mortality rate increased by 25% and 37.5%, respectively. The species exposed to 3.9 *μ*g/mL of extracts were exhibited from the ninth day (12.5% of mortality); on the fourteenth day, the mortality rate increased by 25%. It was only on the tenth day that the mortality (12.5%) of the tadpoles exposed to the control (diclofenac sodium) and to the plant extracts (1.95 *μ*g/mL) was observed.

#### 3.1.3. Description and Relative Proportions of Erythrocytes

**Figure 2:**
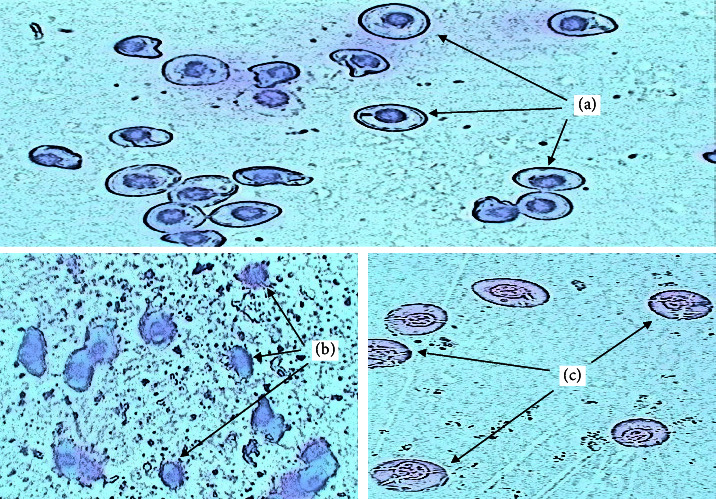
Tadpole frog (*Phrynobatrachus africanus* Hallowell). (a) Mature erythrocytes; (b) erythroblasts; (c) degenerating erythrocytes.

Erythroblasts were characterized by their rounded shape, basophilic cytoplasm, and larger nucleus than that of mature erythrocytes. Besides these cells (Figure 2), degenerating erythrocytes were also observed in some individuals.

**Figure 3:**
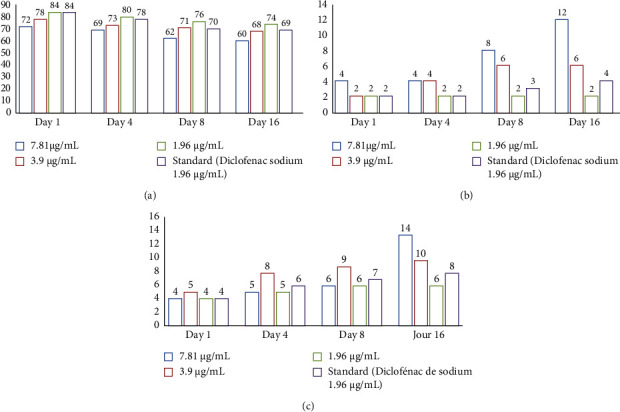
Proportion of erythrocyte cells in the blood of tadpoles exposed to *Antrocaryon klaineanum* for 16 days. (a) Mature erythrocytes; (b) erythroblasts; (c) degenerating erythrocytes.

Figure 3 presents the relative proportions of erythrocytes identified in the blood of tadpoles exposed to extracts of *A. klaineanum* Pierre and diclofenac sodium for 16 days. In Figure 3(a), a relative decrease in mature erythrocytes exposed to plant extracts and diclofenac sodium from the first to the sixteenth day of the study is observed. On the first day, the proportions of mature erythrocytes are 72, 78, 84, and 84%, corresponding to the concentrations of 7.81, 3.9, 1.96, and 1.96 *μ*g/mL (standard), respectively, whereas on day 16, these erythrocytes decrease by 60, 68, 74, and 69%.

The proportions of degenerating erythrocytes in the bloodstream of tadpoles exposed to 7.81, 3.9, and 1.96 *μ*g/mL (Figure 3(c)) are relatively similar, on the first day of exposure (4 to 5%) to that exposed to diclofenac sodium (4%). On the other hand, these proportions rise from 5 to 8% on the fourth day to remain relatively stable until the eighth day and then climb from 6 to 14% on the sixteenth day.

Among the erythroblasts observed (Figure 3(b)), we note their low proportions (2 to 4%) on the first day of exposure to the different concentrations of the plant. From the fourth to the sixteenth day of exposure to 7.81 *μ*g/mL of plant extract, a 4 to 12% increase in erythroblasts is clearly observed. Also, exposure to 3.9 *μ*g/mL of the plant extract shows a relative increase in erythroblasts (4 to 6%) from the fourth to the sixteenth day of the study. Although the proportions of low concentrations do not undergo major changes, it is noted that the proportions of erythroblasts and erythrocytes in degeneration tend to increase with high concentrations and as a function of time.

### 3.2. Antimicrobial Activity of *Antrocaryon klaineanum* Extracts

The results of susceptibility tests with the extracts of *A. klaineanum* gave the following diameters of inhibition on the bacterial strains (Table 2). The screening of the three extracts shows that all have antibacterial activity. The aqueous, water-ethanol, and water-acetone extracts gave the greatest antibacterial activities against *Neisseria gonorrhea* with the inhibition diameters greater than 19 mm. The water-acetone extracts show significant antibacterial activities in the majority of strains with the exception of *E. coli* 105182 CIP, *Salmonella typhi*, *E. coli*, *Staphylococcus aureus*, *Acinetobacter baumannii*, *Enterobacter aerogenes*, and *Salmonella* spp. sensitive to the water-ethanol extract and *Bacillus cereus* LMG 13569 BHI sensitive to the aqueous extract.

The results also show that the fractions possess the greatest antibacterial activities than the crude extracts. These fractions are active against both Gram-positive and Gram-negative bacteria. The Ak F_2_, Ak F_3_, and Ak F_4_ fractions gave the greatest antibacterial activities against the bacterial strains. The Ak F_1_ fraction is active on all strains tested with the exception of *Listeria innocua* LMG 135668 BHI, *Pseudomonas aeruginosa*, and *Salmonella enterica*.

The results of the MICs and CMBs are listed in Table 3. The MICs (0.625 mg/mL) of the water-ethanol extracts (Ak WEE) are the lowest, as are the MICs of the Ak F_2_ and Ak F_3_ fractions.

Table 4 shows the antibacterial effects of crude extracts and fractions of *A. klaineanum*. The aqueous extract has a bactericidal effect on *E. coli* 105182 CIP, *Listeria innocua* LMG 135668 BHI, *Enterococcus faecalis* 103907 CIP, *Bacillus cereus* LMG 13569 BHI, and *Salmonella typhi* strains and has a bacteriostatic effect on *Staphylococcus aureus* ATCC 25293 BHI. The water-ethanol, water-acetone extracts, and the Ak F_2_ fraction have bactericidal actions on all the strains studied. The Ak F_1_, Ak F_3_, and Ak F_4_ fractions exhibit bactericidal effects on the majority of bacteria.

### 3.3. Anti-Inflammatory Activity of *Antrocaryon klaineanum* Extracts

**Figure 4:**
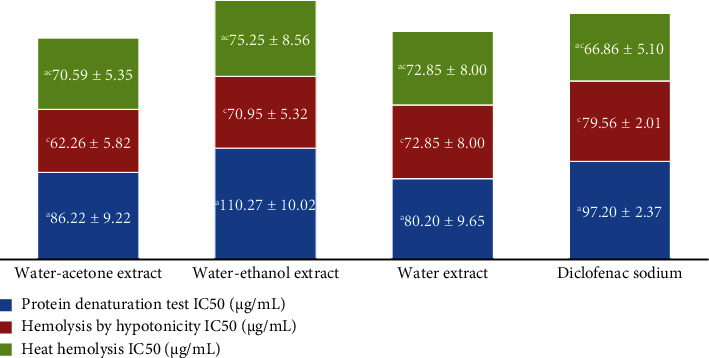
Anti-inflammatory activity of *Antrocaryon klaineanum*.

The results of the in vitro anti-inflammatory activity of the extracts of *A. klaineanum* Pierre are summarized in Figure 4.

In each column, the assigned values of different alphabetic letters (*a*, *b*, *c*) indicate significantly different yields (*P* < 0.05).

This study shows that the water (IC_50_ = 80.20 ± 9.65 *μ*g/mL), water-ethanol (IC_50_ = 110.27 ± 10.02 *μ*g/mL), and water-acetone extracts (IC_50_ = 86.22 ± 9.22 *μ*g/mL) did not show any significant difference compared to the standards (IC_50_ = 97.20 ± 2.37 *μ*g/mL). The hemolysis test induced by a hypotonic solution shows that the three extracts studied presented good activities with IC_50_ ranging from 62.26 ± 5.82 to 72.85 ± 8.00 *μ*g/mL. For heat-induced hemolysis, all extracts exhibited very good antihemolytic activity with IC_50_s ranging from 70.59 ± 5.35 to 75.25 ± 8.56 *μ*g/mL. These results have no significant difference with the standard (diclofenac sodium).

### 3.4. Chromatographic Analyses and Molecular Network MS/MS of *Antrocaryon klaineanum* Pierre Crude Extract

**Figure 5:**
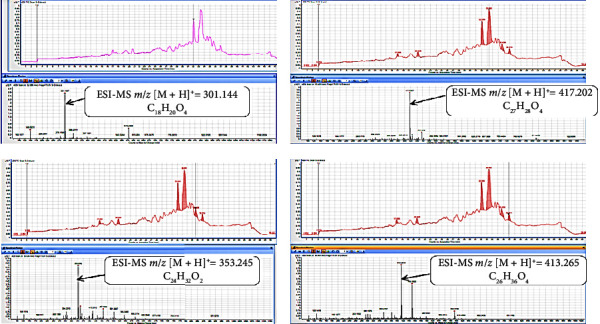
Chromatograms and mass spectra of the ethanol extract of *Antrocaryon klaineanum*.

The results in Figure 5 present a set of chromatogram and mass spectra of the crude extract of *A. klaineanum*. The study reveals 4 major peaks whose masses ESI-MS *m*/*z* [M + H]^+^ = 301.144; ESI-MS *m*/*z* [M + H]^+^ = 353.245; ESI-MS *m*/*z* [M + H]^+^ = 417.202; and ESI-MS *m*/*z* [M + H]^+^ = 413.265 correspond to the respective molecular formulas C_18_H_20_O_4_, C_24_H_32_O_2_, C_27_H_28_O_4_, and C_26_H_36_O_4_. After dereplication of the majority compounds in the databases (Reaxis and Dictionary of natural products), compound 3 (mass ESI-MS *m*/*z* [M + H]^+^ = 353.245) corresponds to monogallate type epicatechin and compound 4 (mass ESI-MS *m*/*z* [M + H]^+^ = 413.256) corresponds to 7*α*-Hydroxy-4,24(28)-ergostadien-3-one (Table 5). The other compounds did not show matches in the literature.

**Figure 6:**
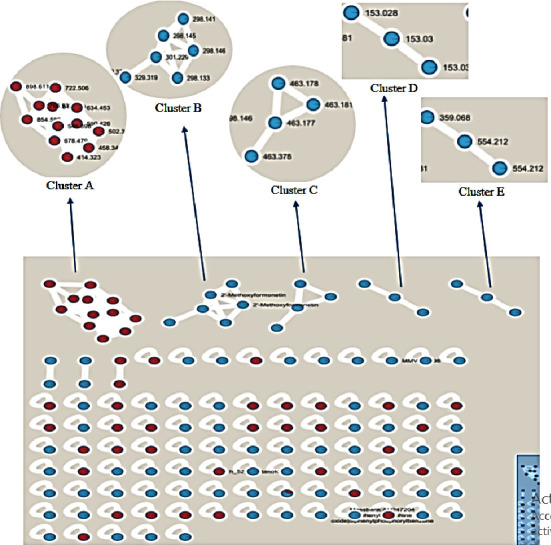
Molecular network of the ethanolic crude extract of *Antrocaryon klaineanum* Pierre.

The results of the molecular network of the crude extract of *A. klaineanum* reveal that the compounds are grouped according to structural similarities and five main clusters emerged (Figure 6): group A presents twelve nodes having the very close molecular masses, group B includes 6 nodes, two masses of which (*m*/*z* 298.145 and 298.146) correspond to Methoxyformonetin. Clusters *C* (4 nodes), *D* (3 nodes), and *E* (3 nodes) group together compounds not identified by our available databases.

## 4. Discussion

The purpose of the work was to study toxicity; establish antibacterial and anti-inflammatory activities, and characterize the phytochemistry of *A. klaineanum* extracts. The results of plant extracts exposed to frog tadpoles show a low toxicity compared to pharmaceutical drugs (diclofenac sodium). The study also shows that the toxicity is progressive over time. This result corroborates with that of Obiang et al. [11], where the authors stipulate that the mortality correlated to the doses used is all the more increased as the exposure of frog tadpoles is prolonged over time. The cells observed on the smears correspond quite closely to the descriptions made in leopard frogs by Jordan [20].

Observation of hematological parameters revealed that exposure of tadpoles to extracts of *A. klaineanum* causes mild anemia. The observed effects were a decrease in the number of mature erythrocytes in the bloodstream, a reduction in erythrocyte length, and rounding of erythrocytes. Several studies presented similar results, which performed experiments on hematological effects in the toad *Bufo regularis* [21]. These authors hypothesized that the appearance of these effects could be associated with an increase in plasma volume; a reduction in blood copper concentration, which plays an important role in erythropoiesis; inhibition of hemoglobin synthesis; and iron deficiency [22]. The essential function of erythrocytes is to ensure the supply of oxygen to the tissues, which is carried, out thanks to hemoglobin. Because of this primary role attributed to hemoglobin, the diagnosis of anemia is only true when there is a decrease in hemoglobin concentration below the lower limit of normality.

The study shows that over time, a slight increase in the proportions of erythroblasts and degenerating erythrocytes was evident. The appearance of young erythrocytes (erythroblasts) reflects a process of renewal of the erythrocyte population. In parallel, degenerating erythrocytes are characteristic of red blood cell lysis. In this case, we could be in the presence of a regenerative type anemia caused by hyperhemolysis of erythrocytes leading to erythropoietic stimulation.

Antibacterial activities of total extracts and fractions of bark extracts of *A. klaineanum* were demonstrated in this study. The results obtained show that each sample tested has inhibitory effects on the growth of the majority of the bacterial strains tested. Of all the bacteria submitted to our study, *Neisseria gonorrhea* showed more sensitivity to the extracts tested. The Ak F_1_, Ak F_2_, and Ak F_3_ fractions proved to be more active on several bacteria compared to the other extracts. These inhibitions may be due to separations and purifications of plant fractions by different chromatography methods [14]. Ethnopharmacological studies have shown that the bark of *A. klaineanum* is used for the treatment of typhoid fever and other liver-related diseases [8]. This study justifies the traditional use of *A. klaineanum* for the fight against certain infections of bacterial origin. The sensitivity of bacteria like *Bacillus cereus* LMG 13569 BHI, *Shigella dysenteria* 5451 CIP, and *Neisseria gonorrhea* to the extracts could direct further research on the therapeutic properties of *A. klaineanum* against gonorrhea, diarrhea, and other types of infections. This sensitivity may be due, in part, to their ability of plant extracts to complex with amino acids of extracellular and soluble proteins resulting in inactivation and/or loss of function.

The anti-inflammatory activity of *A. klaineanum* was evaluated by protein denaturation test and membrane stabilization. Protein denaturation is a process in which proteins lose their tertiary and secondary structures by the application of external stress or compound, such as strong acid, base, or by heat, which most proteins lose their biological function when denatured [23]. Protein denaturation is a well-documented cause of inflammation [19]. The results on inhibition on protein denaturation showed that there is no significant difference between the plant extracts and the reference (diclofenac sodium). Similar results, showing the good inhibitory activities of protein denaturation by medicinal plants have been found in several studies [15, 24].

Stabilization of the red blood cell membrane has been used as a method to study anti-inflammatory activity in vitro because the erythrocyte membrane is analogous to the lysosomal membrane. This method confirms the anti-inflammatory activity of *A. klaineanum* extracts. The results showed that at different concentrations of extracts, erythrocyte membranes were protected against lysis and heat induced by hypotonic solution. Studies have shown that certain plants of the family *A. klaineanum* act at the level of many mechanisms such as the regulation of anti-inflammatory activity. The activation of NF-*κ*B plays a key role in the inflammatory response. These extracts have been shown to reduce the level of production of inflammation mediators such as cyclooxygenase-2 or TNF-*α* [25]. The results of our work confirm the traditional use of *A. klaineanum* in traditional medicine for the treatment of various pathologies [8].

Chromatograms and spectra of *A. klaineanum* show that several major compounds are not part of the dictionary of natural products and other databases consulted with the exception of compounds with masses *m*/*z* [M + H]^+^equal to 417.190 and 353.245. These compounds belong to the polyphenols of the (Epi) catechin-(epi) catechin monogallate type [19] and to the family of ergostane-type steroids [7]. Polyphenols are characterized by the presence of at least one benzene ring on which there is at least one hydroxyl group engaged with other functions. They are known for their antioxidant effects. Polyphenols affect membrane permeability, thus inducing the release of intracellular constituents into the external environment. Moreover, they also interfere with the respiratory chain by inhibiting the oxidation of NADH, the synthesis of nucleic acids and proteins, and the activity of certain enzymes [26].

In addition, we also performed crude extract analysis using the molecular network (MN) approach by GNPS website (https://gnps.ucsd.edu) [27]. All obtained HRESIMS/MS spectra were preprocessed via MZmine2 software following the feature-based molecular networking workflow [28]. Exploring the relative contributions of peak areas to network nodes revealed that compounds were clustered based on structural similarities. The presence of the clusters in the molecular network shows that the crude extract of *A. klaineanum* exhibits several compounds that can be better studied.

## 5. Conclusion

This work made it possible to evaluate the toxicity, the biological (antibacterial and anti-inflammatory) activities, and the chemical characterization of the aqueous extract of the bark of *A. klaineanum*. It emerges from this study that the plant extracts exposed to frog tadpoles show a low toxicity compared to pharmaceutical drugs (diclofenac sodium). Some extracts exhibit bactericidal effects. However, this sensitivity is dose-dependent and varies according to the germs and the extracts. Moreover, the extracts having the good antibacterial properties showed exceptional anti-inflammatory activities, which were significantly very high compared to diclofenac sodium. Phytochemical characterization of the extracts revealed compounds such as monogallate type polyphenols and ergostane-type steroids. This study justifies certain uses of this in traditional medicine.

## Figures and Tables

**Table 1:** Lethal concentration (LC_50_) of aqueous extract *Antrocaryon klaineanum* for frog tadpoles (*Phrynobatrachus africanus* Hallowell).

Species stage of development	LC_50_ (*μ*g/mL) depending on the duration of exposure			
	24 hours	48 hours	72 hours	96 hours
*Phrynobatrachus africanus* Hallowell	110.03 ± 1.25	63.55 ± 4.02	29.36 ± 2.11	15.86 ± 2.21
Tadpoles (2 ± 1 g)	APB	APB	APB	APB
*Rana clamitan*	171.37 ± 9.25	61.99 ± 5.02	56.25 ± 5.01	58.25 ± 7.21
Tadpoles (0.91 g) [17]	EP	EP	REG	REG
*Bufo melanostictu*	22.42	19.81	11.91	8.18
Tadpoles (0.1 g) [18]	EP	EP	REG	REG

APB: appearance and progression of the buds of the hind limbs.

**Table 2:** Inhibition zone diameters produced by the extracts and fractions from *Antrocaryon klaineanum*.

	Inhibition zone diameters (mm)							Standards		
	Extracts			Fractions						
	Ak water	Ak water ethanol	Ak water acetone	Ak F1	Ak F2	Ak F3	Ak F4	Gen	Am	Te
*Bacteria* *Reference strains*										
*Escherichia coli* 105182 CIP	8 ± 1	10 ± 1	9 ± 0	16 ± 2	17 ± 1	16 ± 1	12 ± 1	17 ± 1	Nd	Nd
*Listeria innocua* LMG 135668 BHI	7 ± 0	9 ± 1	11 ± 1	Nd	14 ± 2	13 ± 1	10 ± 0	13 ± 0	7 ± 1	14 ± 0
*Staphylococcus aureus* ATCC 25293 BHI	9 ± 0	10 ± 1	10 ± 0	10 ± 0	12 ± 0	15 ± 0	11 ± 1	15 ± 1	Nd	17 ± 1
*Enterococcus faecalis* 103907 CIP	12 ± 1	11 ± 2	14 ± 0	10 ± 0	12 ± 0	12 ± 2	10 ± 1	30 ± 0	7 ± 1	19 ± 0
*Bacillus cereus* LMG 13569 BHI	10 ± 0	9 ± 1	9 ± 0	15 ± 1	16 ± 0	15 ± 1	11 ± 0	13 ± 1	Nd	18 ± 1
*Shigella dysenteria* 5451 CIP	10 ± 1	10 ± 1	11 ± 1	13 ± 0	15 ± 1	15 ± 0	12 ± 1	24 ± 1	Nd	16 ± 0

*Clinical isolates*										
*Pseudomonas aeruginosa*	7 ± 0	10 ± 1	12 ± 1	Nd	12 ± 1	11 ± 1	9 ± 0	20 ± 0	7 ± 1	21 ± 1
*Salmonella enterica*	8 ± 0	10 ± 1	11 ± 1	Nd	10 ± 0	10 ± 1	11 ± 1	28 ± 1	7 ± 1	16 ± 1
*Salmonella typhi*	9 ± 0	11 ± 1	10 ± 0	9 ± 0	12 ± 1	13 ± 0	10 ± 1	20 ± 1	7 ± 0	15 ± 1
*Neisseria gonorrhea*	19 ± 1	20 ± 1	21 ± 1	21 ± 1	24 ± 0	22 ± 1	18 ± 2	22 ± 2	7 ± 1	10 ± 1

Nd = not determinated; Gen = gentamicin (10 *μ*g/mL), Te = tetracycline (30 *μ*g/mL), Am = ampicillin (30 *μ*g/mL), Ak = *Antrocaryon klaineanum*, and *F* = fraction.

**Table 3:** Minimum inhibitory concentration (MIC) and minimum bactericidal concentration (MBC) or minimum fungicidal concentration (MFC) of crude extracts and fraction of *Antrocaryon klaineanum*.

	MIC and MBC (mg/mL)													
	Ak AE		Ak WEE		Ak WAE		Ak F1		Ak F2		Ak F3		Ak F4	
	MIC	MBC	MIC	MBC	MIC	MBC	MIC	MBC	MIC	MBC	MIC	MBC	MIC	MBC
*Bacteria* *Reference strains*														
*Escherichia coli* 105182 CIP	1.25	2.5	1.25	2.5	1.25	2.5	1.25	1.25	0.625	1.25	1.25	2.5	2.5	5
*Listeria innocua* LMG 135668 BHI	1.25	2.5	1.25	2.5	1.25	2.5	Nd	Nd	1.25	2.5	1.25	5	5	5
*Staphylococcus aureus* ATCC 25293 BHI	1.25	5	1.25	1.25	1.25	2.5	2.5	5	2.5	5	1.25	5	2.5	5
*Enterococcus faecalis* 103907 CIP	1.25	1.25	0.625	0.625	1.25	2.5	5	5	2.5	5	2.5	2.5	5	5
*Bacillus cereus* LMG 13569 BHI	1.25	1.25	0.625	0.625	1.25	1.25	1.25	1.25	0.625	1.25	0.625	1.25	2.5	5
*Shigella dysenteria* 5451 CIP	1.25	2.5	0.625	1.25	1.25	1.25	1.25	5	1.25	2.5	0.625	2.5	>5	>5

*Clinical isolates*														
*Pseudomonas aeruginosa*	1.25	2.5	0.625	1.25	0.625	1.25	Nd	Nd	2.5	5	2.5	5	5	>5
*Salmonella enterica*	2.5	5	1.25	1.25	1.25	1.25	Nd	Nd	2.5	5	5	5	2.5	5
*Salmonella typhi*	2.5	2.5	1.25	2.5	1.25	2.5	5	>5	2.5	5	2.5	2.5	2.5	5
*Neisseria meningitides*	0.625	0.65	0.625	1.25	0.625	1.25	1.25	1.25	0.625	1.25	1.25	1.25	0.625	1.25

Nd = not determinated; Ak = *Antrocaryon klaineanum*, WAE = water-acetone extract; WEE = water-ethanol extract; AE = aqueous extract; *F* = fraction.

**Table 4:** Antimicrobial effects of plants extracts from *Antrocaryon klaineanum*.

	Ak AE	Ak WEE	Ak WAE	Ak F_1_	Ak F_2_	Ak F_3_	Ak F_4_
	MBC/MIC	MBC/MIC	MBC/MIC	MBC/MIC	MBC/MIC	MBC/MIC	MBC/MIC
	Effect	Effect	Effect	Effect	Effect	Effect	Effect
*Bacteria* *Reference strains*							
*Escherichia coli* 105182 CIP	2	2	2	1	2	2	2
*Listeria innocua* LMG 135668 BHI	2	2	2	—	2	3.2	1
*Staphylococcus aureus* ATCC 25293 BHI	4	1	2	2	2	3.2	2
*Enterococcus faecalis* 103907 CIP	1	1	2	1	2	1	1
*Bacillus cereus* LMG 13569 BHI	1	1	1	1	2	2	2
*Shigella dysenteria* 5451 CIP	2	2	1	2	2	2	—

*Clinical isolates*							
*Pseudomonas aeruginosa*	2	2	2	—	2	2	—
*Salmonella enterica*	2	1	1	—	2	1	2
*Salmonella thyphimurium*	1	2	2	—	2	1	2
*Neisseria meningitidis*	1	2	2	1	2	1	2

MBC/MIC < 4: bactericidal; MBC/MIC ≥ 4: bacteriostatic; WAE = water-acetone extract; WEE = water-ethanol extract; AE = aqueous extract.

**Table 5:** HPLC-ESI-QTOF identification of major compounds from ethanol stem barks extracts of *Antrocaryon klaineanum*.

Fraction	Compound	RT	*m*/*z*[M + H]^+^	Score	Formula	Identification DNP	Identification reaxys	Name of compound	Reference
AK Te	1	32.08	301.144	89.31	C_18_ H_20_ O_4_	10 hits	15 hits	Not determined	—
	2	33.40	417.202	70.40	C_27_ H_28_ O_8_	06 hits	7 hits	Not determined	—
	3	35.64	353.245	69.64	C_24_ H_32_ O_2_	32 hits	45 hits	(Epi) catechin-(epi) catechin monogallate (B-type)	[19]
	4	36.86	413.265	80.2	C_26_ H_36_ O_4_	16 hits	56 hits	7a-Hydroxy-4,24(28)-ergostadien-3-one	[6]

AK: *Antrocaryon klaineanum*, Te = total extracts, Rt = retention time, MS = mass spectrometer, DNP = dictionary of natural products.

## Data Availability

The data used to support the findings of this study are included within the article.

## Abbreviations

AK: *Antrocaryon klaineanum*
CM: % corrected mortality
DNP: Dictionary of natural products
EP: Embryonic phase
HPLC: High performance liquid chromatography
IPHAMETRA: Institut de Pharmacopée et de Médecine Traditionennelle
M2: % mortality in the treated population
M1: % mortality in the control population
MBC: Minimum bactericial concentrations
MIC: Minimum inhibitory concentrations
MS: Mass spectrometer
REG: Regression of external gills
Rt: Retention time
TLC: Thin layer chromatography.
